# Longer travel time to district hospital worsens neonatal outcomes: a retrospective cross-sectional study of the effect of delays in receiving emergency cesarean section in Rwanda

**DOI:** 10.1186/s12884-017-1426-1

**Published:** 2017-07-25

**Authors:** Joseph Niyitegeka, Georges Nshimirimana, Allison Silverstein, Jackline Odhiambo, Yihan Lin, Theoneste Nkurunziza, Robert Riviello, Stephen Rulisa, Paulin Banguti, Hema Magge, Martin Macharia, Regis Habimana, Bethany Hedt-Gauthier

**Affiliations:** 1 Department of Anesthesia, Critical Care and Emergency Medicine, School of Medicine and Pharmacy, College of Medicine and Health Sciences, University of Rwanda, P.O. Box 3286, Kigali, Rwanda; 2 Ministry of Health, Kigali, Rwanda; 3 Program in Global Surgery and Social Change, Harvard Medical School, Boston, MA USA; 4 Miller School of Medicine, University of Miami, Miami, FL USA; 5 Partners In Health, Boston, MA USA; 6 Department of Surgery, University of Colorado Hospital, Aurora, CO USA; 7 Partners In Health / Inshuti Mu Buzima, Kigali, Rwanda; 8 Department of Surgery, Brigham and Women’s Hospital, Boston, MA USA; 9 School of Medicine and Pharmacy, College of Medicine and Health Sciences, University of Rwanda, Kigali, Rwanda; 10 Division of Global Health Equity, Brigham and Women’s Hospital, Boston, MA USA; 11 Division of General Pediatrics, Boston Children’s Hospital, Boston, MA USA; 12 Department of Global Health and Social Medicine, Harvard Medical School, Boston, MA USA

**Keywords:** Emergency obstetric care, Rural health delivery, Maternal and newborn health, Neonatal mortality, Quality improvement, Sub-Saharan Africa

## Abstract

**Background:**

In low-resource settings, access to emergency cesarean section is associated with various delays leading to poor neonatal outcomes. In this study, we described the delays a mother faces when needing emergency cesarean delivery and assessed the effect of these delays on neonatal outcomes in Rwanda.

**Methods:**

This retrospective study included 441 neonates and their mothers who underwent emergency cesarean section in 2015 at three district hospitals in Rwanda. Four delays were measured: duration of labor prior to hospital admission, travel time from health center to district hospital, time from admission to surgical incision, and time from decision for emergency cesarean section to surgical incision. Neonatal outcomes were categorized as unfavorable (APGAR <7 at 5 min or death) and favorable (alive and APGAR ≥7 at 5 min). We assessed the relationship between each type of delay and neonatal outcomes using multivariate logistic regression.

**Results:**

In our study, 9.1% (40 out of 401) of neonates had an unfavorable outcome, 38.7% (108 out of 279) of neonates’ mothers labored for 12–24 h before hospital admission, and 44.7% (159 of 356) of mothers were transferred from health centers that required 30–60 min of travel time to reach the district hospital. Furthermore, 48.1% (178 of 370) of cesarean sections started within 5 h after hospital admission and 85.2% (288 of 338) started more than 30 min after the decision for cesarean section was made. Neonatal outcomes were significantly worse among mothers with more than 90 min of travel time from the health center to the district hospital compared to mothers referred from health centers located on the same compound as the hospital (aOR = 5.12, *p* = 0.02). Neonates with cesarean deliveries starting more than 30 min after decision for cesarean section had better outcomes than those starting immediately (aOR = 0.32, *p* = 0.04).

**Conclusions:**

Longer travel time between health center and district hospital was associated with poor neonatal outcomes, highlighting a need to decrease barriers to accessing emergency maternal services. However, longer decision to incision interval posed less risk for adverse neonatal outcome. While this could indicate thorough pre-operative interventions including triage and resuscitation, this relationship should be studied prospectively in the future.

## Background

Limited access to cesarean section contributes to poor neonatal and maternal outcomes worldwide [1–3]. The delays for cesarean delivery can be described using a “three delays” framework – first, the mother’s delay in deciding to seek care; second, the mother’s delay in presenting to the health center or hospital once her decision to seek care is made; and third, the delay in receiving care once at the facility [4, 5]. The negative effects of these delays on maternal and neonatal outcomes are often more pronounced in sub-Saharan Africa [6] due to multiple reasons. Cultural belief that traditional remedies are better than modern medicine, lack of family support, limited financial resources to seek care, lack of health insurance [7–10], substantial distances between the mother’s home and health facilities and inadequate referral systems [7, 10], limited infrastructure to support cesarean deliveries [8–10], and inadequate human resources [8] hinder care seeking behavior.

In sub-Saharan Africa, cesarean section is the most performed major surgery, accounting for 10–50% of all surgical procedures provided at district hospitals [11]. An estimated one in seven cesarean sections in sub-Saharan Africa result in a neonatal death [12]. This is almost ten times greater than the 20 to 22 out of 1000 neonatal deaths following cesarean sections reported worldwide [13]. Among cesarean deliveries, emergency cesarean section requires timely access to and provision of quality care to prevent adverse maternal and neonatal outcomes. Delays in accessing care put both the mother’s and newborn’s lives in danger [2] when complications are not managed on time or worsen due to further delays. This has led to recommendations of timely intervention. For example, the American College of Gynecologists recommends that the decision-to-incision interval should be less than 30 min to promote optimal neonatal outcomes [14]. Although multiple factors can contribute to negative neonatal outcomes [15], understanding which delays drive these outcomes can help focus attention on where preventative interventions are needed.

Rwanda is a low-income country in sub-Saharan Africa, with a neonatal mortality ratio of 20 per 1000 live births [16]. The maternal mortality ratio has decreased considerably, but is not parallel to neonatal mortality, which continues to be high [16]. Several initiatives to improve obstetrical and neonatal care in the country, such as advocacy for facility delivery, new infrastructure for delivery and neonatal services, and capacity strengthening for health providers through the Human Resources for Health training program, are ongoing [16]. Between 2010 and 2015, deliveries by skilled health providers improved from 69% to 90% [16]. In Rwanda, 13.0–14.7% of births in 2014 were delivered by cesarean section [16, 17]. More than 60% of the operations performed at rural district hospitals in the country are cesarean sections [18]. Similar to other low-resource settings, Rwandese mothers experience delays in obtaining cesarean section [8]. This study described the delays pregnant women face when a decision for cesarean delivery was made, and assessed the effect of the delays on neonatal outcomes at three rural district hospitals in Rwanda.

## Methods

### Study design and setting

This retrospective cross sectional study was conducted at three rural district hospitals (Butaro, Kirehe and Rwinkwavu) in Rwanda. These district hospitals are under the management of the Rwandan Ministry of Health (MOH) with technical and operational support provided by an international non-governmental organization, Partners In Health, known locally as Inshuti Mu Buzima (PIH/IMB). These district hospitals receive patients referred from 46 health centers in their catchment area. At each of the district hospitals, there are two operating rooms where cesarean deliveries can be performed and most cesarean deliveries are conducted by general practitioners.

In Rwanda, pregnant women and their families are responsible for their transport from home to the health center in their catchment area. Sometimes community health workers accompany laboring women to the health center. At the health center, registered nurses or midwives manage the woman. In the event of a potential complication for the fetus or the woman, the nurses/midwives will transfer the woman to the nearest district hospital via ambulance or with transport arranged by the woman’s family if no ambulance is available. Each district hospital has five ambulances for all emergencies, while only 11 out of the 46 health centers in the districts of study have ambulances. The rest of the health centers depend on ambulances from the district hospital for emergency transport. Most of the roads in these rural settings are unpaved and public transport has limited reliability. In cases where the health center is located in the same compound as the district hospital, an ambulance is not necessary for the transport. On rare occasions, laboring women present directly to the district hospital.

At the district hospital, registered nurses or midwives perform initial obstetrical review and call general practitioners for further laboratory and ultrasound assessments, and decision for cesarean delivery. If cesarean delivery is decided, the pregnant woman is transferred to a delivery room where nurse-anesthetists administer anesthesia and the general practitioner, supported by nurses or midwives, performs cesarean section. Each hospital assists with 10–12 deliveries per day. During the day, one general practitioner is assigned to the delivery room; at night, one general practitioner covers the entirety of the maternity department. At all times, two nurses or midwives are assigned to the delivery room. After delivery, the woman and the neonate are transferred to the post-partum room where midwives closely monitor their progress with daily check-ups by the general practitioners and until discharge. Obstetricians are sometimes but not always available at the district hospitals.

The decision to seek care at a health facility depends on the ability to pay or having a valid health insurance [16]. The majority of Rwandan households (79%) have at least one member with health insurance [16]. For women who have community-based health insurance (97% of households with insurance [16]), the insurance covers 90% of the total cost of care and the woman pays the remaining balance. Women without health insurance cover entirety of the cost out of pocket.

### Study population

The study included mothers and their neonates born via emergency cesarean section between 01 January 2015 and 31 December 2015 at the three hospitals. All emergency cesarean sections without intrauterine fetal death prior to the decision for cesarean section were eligible for inclusion. Because of the large population of cesarean deliveries at the district hospitals, we calculated a study sample size that could detect differences in neonatal outcomes by delays. Due to limited data a priori*,* we assumed that the overall negative neonatal outcome was 50% and that delay categories were binary to calculate the largest sample size possible. To have a 90% power to detect a 20% difference in neonatal outcome (i.e. one group had 40% negative neonatal outcome and the second group had 60% negative neonatal outcome), with α=0.05, we calculated a necessary sample of 140 women per group (delayed or not delayed). In anticipation of missing data, we increased the sample size by 30% to 200 per group or 400 women total. We further increased the sample size to 600 women, 200 per hospital, to account for any losses in statistical efficiency due to stratification by hospital and differences in group sizes.

As the majority of cesarean deliveries at these hospitals were due to the referral of emergent and complicated cases from health centers, we originally assumed all cesarean deliveries were emergency and used segmented sampling to sample 200 women in each hospital. However, if a woman’s chart did not meet the inclusion criteria, that is if the cesarean delivery was indicated as elective or the infant died before the decision for cesarean section, we removed and replaced the chart with an eligible chart, randomly picked from the same month of admission. Among the sampled cesarean section deliveries, we performed in-depth review of indications for cesarean section. During the data cleaning stage, we excluded any deliveries whose indication for cesarean section was not emergent. These included women whose sole indication for cesarean delivery was previous cesarean delivery, women who were past their due date, or women who were having twins without any other emergent indication. Included emergency cesarean deliveries had at least one of the following indications: cord prolapse, uterine rupture with a previous cesarean section scar, fetal distress, eclampsia, obstructed labor, mal-presentation, cephalopelvic disproportion, prolonged rupture of membranes with a previous cesarean section scar, and pre-eclampsia [19, 20]. Finally, any neonate who did not have record of neonatal outcome, either death within the first 24 h or APGAR at 5 min after delivery was excluded from analysis.

### Data collection and definitions

Trained data collectors extracted chart data on women’s demographic and clinical characteristics, individual and health facility delays, and neonatal outcomes. We categorized the indication for cesarean delivery as very severe or severe through consultation with a local obstetrician and based on complications to the fetus. Very severe indications included fetal distress, cord prolapse, uterine rupture, eclampsia, abruption placenta, and obstructed labor. Severe indications included prolonged rupture of membranes, pre-eclampsia, placenta previa, cephalopelvic disproportion, and mal-presentation, including breech presentation, transverse presentation, and occiput posterior presentation. If a woman had more than one indication, we prioritized the most severe indication. A neonatal outcome was considered favorable if the neonate was alive and had an APGAR score ≥ 7 at 5 min and unfavorable if there was death within 24 h after delivery or an APGAR score < 7 at 5 min.

**Fig. 1:**
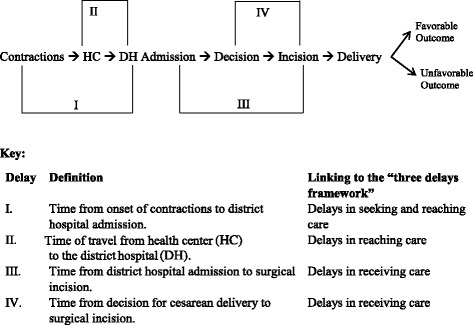
Possible delays for emergency cesarean section

We captured four measures of delays in reaching and receiving care that were routinely collected in the charts (Fig. 1). We used two measures for delays in reaching care. First, we looked at the duration of labor prior to district hospital admission, as self-reported by the women. We also used the average ambulance travel time from the health center, where an emergency condition occurred or could have been detected, to the district hospital. The latter was a standardized time based on health center records and did not include the actual time from when the emergency was detected until the ambulance was called or the actual travel time for the mother in case an ambulance was not available. We used three cutoffs for this measure: less than 30 min (indicating that the health center was located on the same compound as the district hospital), 30–60 min, and more than 60 min. Delays in receiving care were measured by the time from admission to the district hospital to surgery start and the time from the decision for a cesarean section by a doctor to surgical incision (decision-to-incision interval).

### Data analysis

**Fig. 2:**
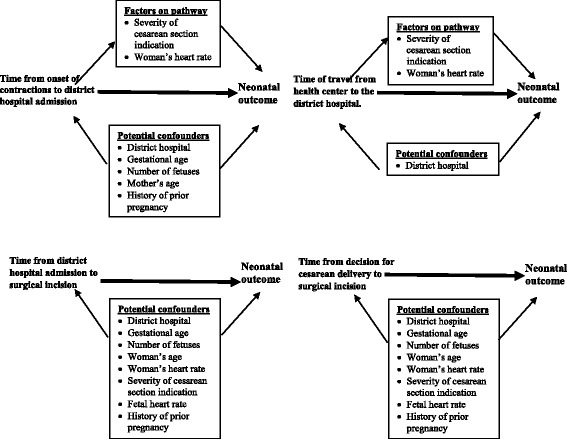
Directed acyclic graphs (DAGs) mapping the relationship between the four possible delays and neonatal outcomes, noting potential confounders

We assessed the relationship between neonatal outcome and potential confounders using Chi-squared tests. Types of delays were described using frequencies. We assessed the relationship between each of the delays and the neonatal outcome using multivariate logistic regression, controlling for potential confounders. We developed a separate model for each delay predictor. Potential confounders considered for this study were mother’s age, gestational age, number of fetuses, woman’s heart rate, fetal heart rate, district hospital, history of prior pregnancy, and severity of indication. To determine the appropriate confounders for each delay and neonatal outcome, i.e. the specific factors that were potentially related to the delay and the outcome but were not on the causal pathway, we constructed directed acyclic graphs (DAGs) (Fig. 2). For the duration of labor prior to admission at the district hospital, we controlled for district hospital, gestational age, number of fetuses, history of prior pregnancy, and woman’s age as potential confounders. For the travel time from health center to district hospital, we controlled for district hospital as a potential confounder. For the admission to surgical incision and decision to incision intervals, we controlled for district hospital, gestational age, number of fetuses, woman’s age, woman’s heart rate, fetal heart rate, and severity of cesarean section indication. We reported the resulting odds ratios (OR), adjusted odds ratios (aOR), 95% confidence intervals (95% CIs), and *p*-values.

For bivariate analyses, we excluded all observations with missing data. We also excluded observations if delay for that individual was not measured; however, if a confounder was included in a multivariate analysis, we created a missing category for that confounder if more than 15% of patients had missing data in order to avoid excluding these patients from the analysis. We used α = 0.05 significance level for all analyses and completed all analyses using Stata v14 (College Station, TX: StataCorp LP).

## Results

**Fig. 3:**
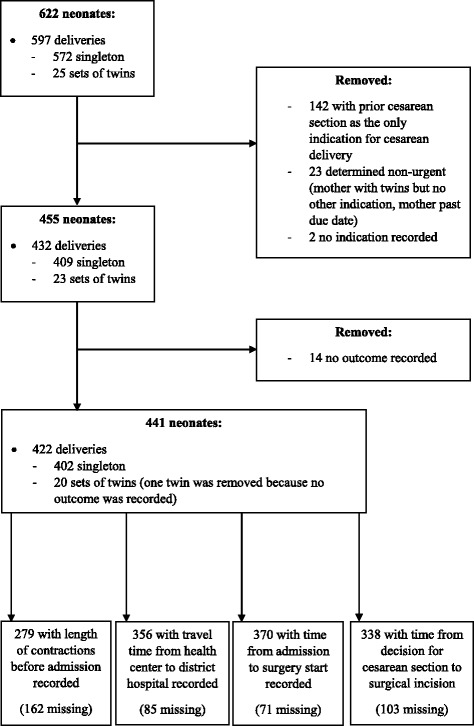
Study inclusion criteria

In 2015, there were 2339 emergency cesarean section deliveries in the three district hospitals, of which we sampled 597 deliveries corresponding to 622 neonates (Fig. 3). Of the 622 neonates, we confirmed that 455 had emergent indications for their cesarean sections. An additional 14 were excluded due to lack of neonatal outcome records, resulting in 441 neonates included in the analysis. Of these, 401 (90.9%) survived and had APGAR score ≥7 at 5 min while 40 (9.1%) died or had five-minute APGAR score <7 (Table 1). Overall, 13 (2.9%) neonates died, 27 (6.1%) had APGAR scores <7, and 14 (51.9%) of those with APGAR scores <7 were transferred or admitted to neonatology unit. Among the potential confounders, most had no significant association with the neonatal outcome with *p* > 0.10 for district hospital, mother’s age, gestational age, number of fetuses, systolic blood pressure, maternal heart rate, and fetal heart rate. However, there was a trend between severity of indication for cesarean section and outcome, as very severe indications had a higher rate of neonatal deaths or low APGAR scores compared to severe indications (12.2% vs. 6.9%, *p* = 0.06).

**Table 1:** The effect of demographic factors on neonatal outcomes post emergency cesarean section in Rwanda (*N* = 441)

	Survived and APGAR ≥7		Died or APGAR <7		
	*n*	*%*	*n*	*%*	*p-value*
Overall	401	90.9	40	9.1	N/A
Neonatal death	-	-	13	3.0	N/A
Neonates with APGAR <7	-	-	27	6.1	N/A
District hospital					
Butaro	147	91.3	14	8.7	0.47
Kirehe	129	92.8	10	7.2	
Rwinkwavu	125	88.7	16	11.3	
Woman’s age (years) (*N* = 436)					
15–24	163	92.6	13	7.4	0.41
25–34	171	90.5	18	9.5	
35–44	62	87.3	9	12.7	
Gestational age (weeks) (*N* = 366)					
< 37	35	92.1	3	7.9	0.88
37–41	247	92.5	20	7.5	
> 41	56	91.8	5	8.2	
Number of fetuses					
1	368	91.5	34	8.5	0.15
2	33	84.6	6	15.4	
Indication for cesarean section					
Very severe^a^	158	87.8	22	12.2	0.06
Severe^b^	243	93.1	18	6.9	
Systolic blood pressure (*N* = 404)					
< 90	3	100.0	0.0	0.0	0.85
90–140	354	91.5	33	8.5	
> 140	13	92.9	1	7.1	
Maternal heart rate (beats per minute) (*N* = 383)					
≤ 100	311	92.3	26	7.7	0.46
> 100	41	89.1	5	10.9	
Fetal heart rate (beats per minute) (*N* = 429)					
< 120	54	91.5	5	8.5	0.46
120–160	323	91.8	29	8.2	
>160	15	83.3	3	16.7	

^a^Very severe indication: intrauterine rupture, fetal distress, cord prolapse, abruption placenta

^b^Severe indication: preeclampsia, prolonged rupture of membranes, cephalopelvic disproportion, prolonged labor and mal-presentation

In assessing rates of delay, among 279 neonates whose mother’s duration of labor was recorded, the mothers of 108 (38.7%) neonates were in labor for 12-24 h prior to admission at the district hospital (Table 2). For the 356 neonates whose mother’s ambulance travel time from health center to district hospital was calculated, 159 (44.7%) were from health centers located 30-60 min away from the district hospital. Cesarean deliveries were started within 5 h after admission in 178 out of 370 cases (48.1%). In an additional 100 (27%) cases, the operation started more than 15 h after admission. For the majority of cesarean sections (*n* = 288 out of 338, 85.2%), the decision to incision interval was greater than 30 min.

**Table 2:** Time delays for emergency cesarean section in Rwanda

Type of Delay	*n*	*%*
Duration of labor before hospital admission (hours) (*N* = 279)		
< 12	83	29.7
12 to <24	108	38.7
24 to <36	46	16.5
≥ 36	42	15.1
Travel time from HC to DH (minutes) (*N* = 356)		
HC located on the same compound as the DH (<30)	91	25.6
30 to <60	159	44.7
60 to <90	45	12.6
≥ 90	61	17.1
Time from admission to surgery (hours) (*N* = 370)		
≤ 5	178	48.1
> 5 to ≤10	55	14.9
> 10 to ≤15	37	10.0
> 15	100	27.0
Decision to delivery interval (minutes) (*N* = 338)		
< 30	50	14.8
≥ 30	288	85.2

*HC* Health Center

*DH* District Hospital

In the unadjusted model, both the duration of labor prior to admission as well as the varying times between admission and surgery start were not significantly linked to neonatal outcome (*p* > 0.05 for all categories). The shorter decision to incision interval had a trend towards a negative neonatal outcome (*p* = 0.09). Longer ambulance travel time from health center to district hospital had a strong association with poor neonatal outcome (*p* = 0.07 for 30–60 min, *p* = 0.04 for 60–90 min and *p* = 0.01 for 90+ minutes compared to the individuals from health centers located on the same compound as the district hospital).

After adjusting for potential confounders, there was no statistically significant association between duration of labor before hospital admission and time from admission to surgery with neonatal outcome. Compared to less than 12 h of labor prior to arrival, the adjusted odds ratio (aOR) for neonatal death or low APGAR score for deliveries within 12-24 h, 24-36 h and 36+ h of labor were ≤1.20 (*p* > 0.05 for all categories) (Table 3). Compared to the time from admission to surgery of <5 h, time from admission to surgery of 5–10 h and 10-15 h both had an aOR ≥1.12 (*p* ≥ 0.08) and time from admission to surgery >15 h had an aOR of 0.50 (*p* = 0.35).

**Table 3:** The effect of time delays for emergency cesarean section on neonatal outcomes in Rwanda

	Unadjusted			Adjusted		
	OR	*p*-value	95% CI	aOR	*p*-value	95% CI
Duration of labor before hospital admission (hours)^a^						
< 12	ref	-	-	ref	-	-
12 to <24	0.65	0.42	[0.23, 1.87]	0.81	0.71	[0.26, 2.50]
24 to <36	0.43	0.29	[0.09, 2.10]	0.54	0.38	[0.10, 2.92]
≥ 36	0.99	0.98	[0.27, 3.49]	1.20	0.80	[0.31, 4.65]
Travel time from HC to DH (minutes)^b^						
HC located on the same compound as the DH (<30)	ref	-	-	ref	-	-
30 to <60	3.28	0.07	[0.93, 11.59]	3.02	0.09	[0.84, 10.84]
60 to <90	4.51	0.04	[1.07, 18.98]	4.31	0.05	[1.02, 18.29]
≥ 90	5.75	0.01	[1.51, 21.87]	5.12	0.02	[1.30, 20.21]
Time from admission to surgery (hours)^c^						
≤ 5	ref	-	-	ref	-	-
> 5 to ≤10	1.85	0.22	[0.70, 4.90]	3.00	0.08	[0.89, 10.08]
> 10 to ≤15	0.73	0.68	[0.16, 3.36]	1.12	0.89	[0.20, 6.15]
> 15	0.81	0.68	[0.30, 2.20]	0.50	0.35	[0.12, 2.10]
Decision to incision interval (minutes)^c^						
< 30	ref	-	-	ref	-	-
≥ 30	0.48	0.09	[0.20, 1.13]	0.32	0.04	[0.11, 0.96]

*Ref* Reference value

*HC* Health Center

*DH* District Hospital

*OR* Odds Ratio

*aOR* adjusted Odds Ratio

^a^Adjusted for district hospital, gestational age, number of fetuses, and woman’s age

^b^Adjusted for district hospital

^c^Adjusted for district hospital, gestational age, number of fetuses, woman’s age, woman’s heart rate, severity of cesarean section indication, and fetal heart rate

However, while adjusting for confounders, a longer travel time from the health center to the district hospital remained significantly associated with death or low APGAR score. Compared to travel time from health centers located on the same compound as the district hospital, travel time of 30–60 min had an aOR of 3.02 (95% CI: 0.84, 10.84), travel time of 60–90 min had an aOR of 4.31 (95% CI: 1.02, 18.29), and travel time of more than 90 min had an aOR of 5.12 (95% CI: 1.30, 20.21). For the decision to incision interval, neonates whose mothers had a decision to incision interval of 30 min or more were significantly less likely to die or have low APGAR scores (aOR = 0.32, 95% CI: 0.11, 0.96). In analysis not shown in the tables, 13 deliveries had intra-operative complications, 11 of which were bleeding. Of the 13 complications, ten occurred with a decision to incision interval of <60 min (three for <30 min and seven for 30–60 min), and three with a decision to incision interval ≥ 60 min.

## Discussion

In this study, we found that pregnant women experience diverse and considerable delays from the initiation of labor to the start of an emergency cesarean section. Nearly a third of women had labored for over 24 h before arriving at the district hospital. Of note, most women first go to their catchment health center to seek care and are only referred to the district hospital once an emergency occurs [21]. If they are referred, the travel time from the health center to the district hospital was often 30 min or more. This is likely an underestimation of the actual time since our study did not capture the real-time duration between when an emergency was detected until the woman reached the district hospital. More delays can occur when a woman waits for the ambulance to arrive or when she has to arrange her own transport if an ambulance is not available. These prehospital delays have previously been attributed to delays in deciding to seek healthcare due to limited finances or lack of family support [7–10, 16], underestimation of the severity of complication, previous poor experience with the health care system, lack of or prolonged transport, and seeking care in many health facilities that lack the needed capacity [1, 16, 22].

Additional delays in receiving care occur between a woman’s presentation at the health center and her arrival at the district hospital. At the health center, lack of ambulances for emergency transport or insufficient ambulance staff delay referral to the district hospital. After admission to the district hospital, midwives and registered nurses monitor women, while the general practitioner assesses and determines the need for an emergency cesarean section. The majority of women in our study received surgery more than 5 h after admission. Furthermore, most women received surgery more than 30 min after a general practitioner had decided that she needed a cesarean section. This delay, which could be due to too few providers, inadequate medication and equipment, and too few operating rooms [8–10], is consistent with other studies in sub-Saharan Africa, suggesting a 30-min decision to incision interval may not be realistic in low- or middle-income settings [23]. Of note, there could also be a potential unmeasured delay between nurse notification and doctor assessment, which was not captured systematically in the patient files for inclusion in this study but should be assessed in future studies.

While delayed emergency cesarean section is often linked to poor neonatal outcomes [3], two of the delays we considered, the duration of labor prior to admission to the district hospital and the time from admission to surgery start, were not significantly associated with neonatal death or low APGAR scores. However, we found a significant association between ambulance travel time from the health center to the district hospital, with longer travel time associated with higher odds for neonatal death or low APGAR scores. This is consistent with other studies that link poor maternal and neonatal outcomes to distance traveled by the woman before she reaches the hospital [24]. In Rwanda, we believe that this effect is compounded when there are no ambulances available to transport a laboring woman to the district hospital. For the 46 health facilities in the catchment area of the district hospitals included in this study, only 11 have an ambulance available at the facility and the remainder request for an ambulance to be dispatched from another location. Increasing timely access from health centers to the district hospital, either by adding more district hospitals or by increasing the availability of ambulances, will likely result in better neonatal outcomes after emergency cesarean delivery.

While some studies in sub-Saharan Africa have not reported a link between the decision to incision interval and neonatal outcomes [25], the prevailing consensus is that a shorter interval is associated with improved neonatal outcomes [14], with a 30 min target commonly set for the decision to incision interval [23, 26]. To our surprise, we found that neonates born with a longer decision to incision interval had better outcomes. We propose possible hypotheses that could explain this result. First, this may be due to triaging by hospital staff, where patients who were determined to be less emergent and therefore more likely to have better outcomes, are less likely to go to the operating theatre immediately. Such a phenomenon is a noted confounder in a meta-analysis on this topic [27]. However, a second explanation could be that rushing the surgery may lead to less than ideal surgical conditions, for example, due to a lack of optimization of the patient or inadequate materials due to lack of preparation. Complications from emergency cesarean section include sepsis, wound dehiscence, anemia, and others [28]. It is also possible that some indications for cesarean section such as fetal distress were not actually present but were documented to reflect a decision to perform a cesarean section. Further studies related to the quality of care are needed to explore the relationship between decision to incision interval and neonatal outcome in this context.

Our study has several limitations to consider. First, we used routinely collected clinical data, and as a result, some data were incomplete. However, because we expected to have missing data, we accounted for this when calculating our minimum sample size. For all of the women whom we collected data on, the majority (280 patients) had information on major delays, which was adequate to detect a difference in outcomes. Second, given that this study was only conducted at three district hospitals, all of which receive some support from Partners In Health, the results may have limited generalizability, as there may be variations in staffing and supply chain at these hospitals. However, the Rwandan Ministry of Health manages all three public hospitals, with the same standardized structures in place as other district hospitals throughout Rwanda. Therefore, we believe our findings to be representative of the Rwandan rural setting and may be similar to other rural district hospitals in the region. Additionally, since time estimates relied on information existing in the medical charts, we were not able to measure some delays, such as delay in deciding to seek care, the actual travel time from health center to district hospital, or the time between nurse or midwife notification for cesarean section assessment by doctor and doctor assessment and decision for cesarean delivery. Furthermore, some of the time estimates such as duration of labor before hospital admission were self-reported by the women and thus subject to recall bias. The self-reported time data did not capture moments such as when labor began, when decision to seek care was made, and when initial care was sought. We recommend future prospective studies to tease out delays in seeking care and factors leading to these delays, which are important in improving maternal and neonatal outcomes in this setting. Finally, as observed in a previous systematic review [19], the definitions of cesarean section indications such as fetal distress were ambiguous and our classification of cesarean sections as emergency or as severe and very severe were subjective, limiting generalizability. In addition, due to time and resource constrains, we did not report outcomes for neonates admitted to neonatology units. A prospective audit of cesarean section rates in Rwanda should provide more evidence on the categorizations for urgency of cesarean section, and follow neonates admitted to neonatology units to report outcomes.

## Conclusions

Our study found that a longer travel time from health centers to the district hospital was associated with adverse neonatal outcomes. This emphasizes the need for strategies to reduce the transfer delay from health centers to district hospitals, including improving detection of maternal/fetal complications and response time, referral systems, improving road networks, increasing the number of ambulances and district hospitals, and subsidizing transport costs. However, longer time lag from a decision to perform an emergency cesarean section to the surgical incision was associated with less risk of poor neonatal outcome possibly due to an opportunity of thorough pre-operative interventions including resuscitation and triage. The achievability of a 30-min decision to incision interval and the relationship between this interval and neonatal outcomes requires further prospective studies to make appropriate recommendations for this context and investigate factors leading to delays that worsen neonatal outcomes.

## Abbreviations

95% CI: 95% Confidence Interval
aOR: Adjusted Odds Ratio
APGAR: Appearance, Pulse, Grimace, Activity and Respiration
DAGS: Directed acyclic graphs
OR: Odds Ratio
